# Preimplantation genetic testing for BRCA gene mutation carriers: a cost effectiveness analysis

**DOI:** 10.1186/s12958-021-00827-9

**Published:** 2021-10-08

**Authors:** Nadav Michaan, Moshe Leshno, Yoni Cohen, Tamar Safra, Shira Peleg-Hasson, Ido Laskov, Dan Grisaru

**Affiliations:** 1 Gynecologic Oncology Department, Lis Maternity Hospital, Tel Aviv Sourasky Medical Center, Sackler School of Medicine, Tel Aviv University, 6 Weismann st., 6296317 Tel Aviv, Israel; 2 Gastro-enterology, Tel Aviv Sourasky Medical Center, Coller School of Management and Sackler School of Medicine, Tel Aviv University, Tel Aviv, Israel; 3 In-vitro Fertilization Unit, Lis Maternity Hospital, Tel Aviv Sourasky Medical Center, Sackler School of Medicine, Tel Aviv University, Tel Aviv, Israel; 4 Oncology Department, Tel Aviv Sourasky Medical Center, Sackler School of Medicine, Tel Aviv University, Tel Aviv, Israel

**Keywords:** IFV/PGT-M, BRCA, Cost-effectiveness, Ovarian cancer, Breast cancer

## Abstract

**Background:**

Gynecologic oncologists should be aware of the option of conception through IVF/PGT-M for families with high BRCA related morbidity or mortality. Our objective was to investigate the cost-effectiveness of preimplantation genetic testing for selection and transfer of BRCA negative embryo in BRCA mutation carriers compared to natural conception.

**Methods:**

Cost-effectiveness of two strategies, conception through IVF/PGT-M and BRCA negative embryo transfer versus natural conception with a 50% chance of BRCA positive newborn for BRCA mutation carriers was compared using a Markovian process decision analysis model. Costs of the two strategies were compared using quality adjusted life years (QALYs’). All costs were discounted at 3%. Incremental cost effectiveness ratio (ICER) compared to willingness to pay threshold was used for cost-effectiveness analysis.

**Results:**

IVF/ PGT-M is cost-effective with an ICER of 150,219 new Israeli Shekels, per QALY gained (equivalent to 44,480 USD), at a 3% discount rate.

**Conclusions:**

IVF/ PGT-M and BRCA negative embryo transfer compared to natural conception among BRCA positive parents is cost effective and may be offered for selected couples with high BRCA mutation related morbidity or mortality. Our results could impact decisions regarding conception among BRCA positive couples and health care providers.

**Supplementary Information:**

The online version contains supplementary material available at 10.1186/s12958-021-00827-9.

## Background

BRCA mutation carriers have an estimated 80% life time risk of breast cancer, up to 40% life time risk of ovarian cancer, as well as increased risk of other malignancies including gastro-intestinal, pancreatic and prostate cancer [1]. In some family clusters, high disease burden is noticed with several generations effected at young ages, causing significant physical and psychological morbidity [2, 3]. Previous studies have shown that population based BRCA screening is cost effective and can be used as a screening tool that allows very effective risk reduction strategies for BRCA carriers [4–6]. Another possible strategy that may prevent passing on the BRCA gene to next generations is selection of BRCA negative embryos using in-vitro fertilization (IVF) and preimplantation genetic testing for monogenic/single gene disorders (PGT-M). PGT-M enables selection of unaffected embryos for embryo transfer and may be used for prevention of single gene disorders, such as BRCA gene mutations, in offspring [7, 8]. The Ethics committee opinion of the European Society of Human Reproduction and Embryology stated that PGT-M for adult-onset conditions is ethically justifiable when the conditions are serious and when there are no known interventions for the conditions, or the available interventions are either inadequately effective or are perceived to be significantly burdensome [9]. Indeed, delivering a BRCA negative newborn would prevent the need for life long cancer surveillance for BRCA positive patients along with the medical, psychological and financial burden associated, and may be a suitable solution for some BRCA positive families.

The aim of our study was to investigate whether IVF/ PGT-M for BRCA negative embryo selection among BRCA positive parents as opposed to natural conception with a 50% chance of a BRCA positive fetus, due to dominant gene inheritance, would be a cost effective strategy.

## Methods

**Fig. 1:**
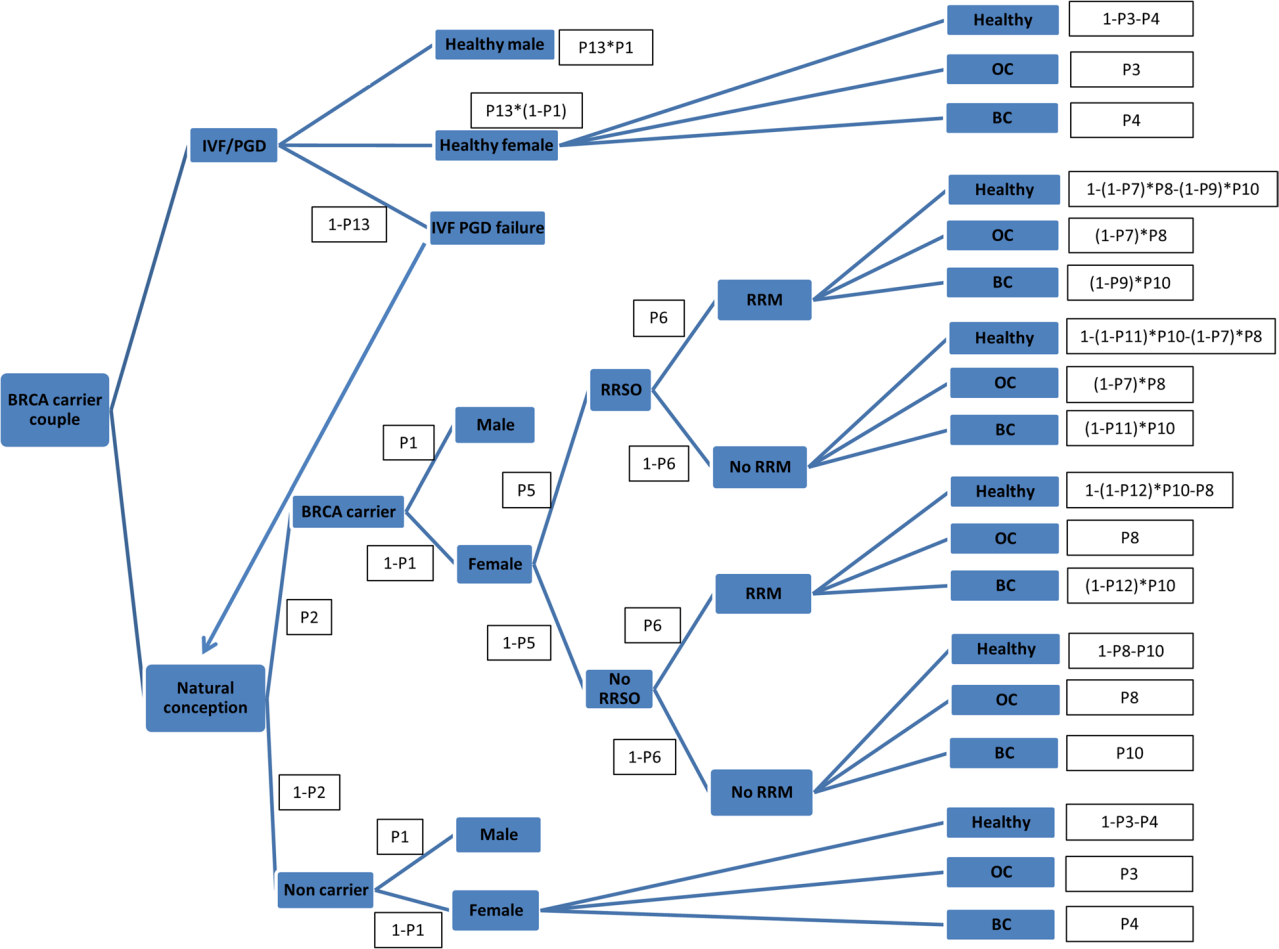
Markovian decision analysis model, IFV/PGD for BRCA mutation carriers versus natural conception

The target population for our research are potential BRCA positive parents (mother or father). Costs of IVF/PGT-M, with BRCA negative embryo selection and transfer versus natural conception with a 50% chance of BRCA positive newborn, were compared using a Markovian process decision analysis model (Fig. 1). The model assumed that all women in the IVF/ PGT-M arm would undergo ovarian stimulation and ovum pick. After intracytoplasmic sperm injection (ICSI), fertilization and embryo biopsy, a BRCA negative embryo would be selected for embryo transfer. In the natural conception arm, women would conceive naturally, without any manipulation, and assume a 50% chance of bearing a BRCA positive newborn, as BRCA is a dominant gene. BRCA negative newborns would assume to have the general populations’ life-time-risk of breast and ovarian cancer. At age 40, BRCA positive females would be offered risk reduction salpingo-oophorectomy (RRSO) for ovarian cancer prevention [10]. Management of breast cancer risk for BRCA positive females would include screening with yearly breast MRI/ultrasound or risk reduction mastectomy (RRM) for breast cancer prevention [10]. Costs of these two strategies were compared using quality-adjusted life years (QALYs), which reflect both quality and quantity of life lived. Incremental cost effectiveness ratio (ICER) was used for cost-effectiveness analysis, compared to a willingness to pay threshold.

### IVF/PGT-M arm

In this arm, all couples with either one BRCA positive parent were assumed to undergo ovarian stimulation, ovum pick-up and ICSI. After fertilization, embryos would be biopsied and only BRCA negative embryos would be transferred. All women were assumed to have IVF before age 35, where IVF success rates are optimal [11], as the knowledge of BRCA carrier state among afflicted families would to be known at an early age. The following steps were assumed in the IVF/ PGT-M arm, probabilities were taken from the ESHRE PGD consortium data collection regarding success of IVF/ PGT-M cycles preformed for single, autosomal dominant gene disorders [8]: a: ovarian stimulation and ovum pick-up, on average, 13 oocytes are retrieved per IVF cycle among patients< 35 years, undergoing IVF/PGD for dominant, single gene disorders, b: insemination with ICSI, 82% of oocytes are successfully inseminated, c: fertilization,76% of inseminated oocytes are fertilized, d: embryo biopsy, 79% of embryos are successfully biopsied.

Accordingly, per cycle start, out of 13 oocytes retrieved, 6.4 embryos will be available for biopsy, half of which, 3.20 embryos, will be BRCA negative embryos, available for transfer. The first embryo will be used for fresh embryo transfer, the remaining embryos will be frozen for future frozen thawed embryo transfer cycles.

The overall live birth rate per embryo transfer for couples undergoing IVF/ PGT-M for genetic disorders reaches 45.8% [12]. The model assumed that after the first fresh embryo transfer, 54.2% of couples who fail would have a second thawed embryo transfer, while 29% of couples who will fail the second transfer, will have a third, thawed embryo transfer. Therefore, 3 available healthy, non BRCA mutated embryos, with a 45.8% live birth rate per embryo transfer would result in an overall 84% chance of a live, BRCA negative baby, from one cycle of fresh embryo transfer and two more cycles of thawed embryo transfers. The remaining 16% of couples who failed the IVF/ PGT-M path would go back to the natural conception arm.

### Natural conception arm

In this arm, couples are assumed to conceive naturally. As BRCA is a dominant gene, these couples would have a 50% chance of bearing a BRCA positive fetus, of those, 50% would be females, positive for BRCA gene mutations with respective increased risk of breast and ovarian cancer. As the chances of other BRCA related malignancies among male mutation carriers are much lower than among female mutation carriers these were not included in our model. BRCA negative newborns were assumed to have the natural populations’ breast and ovarian cancer risk.

### Model costs

In vitro fertilization, PGT-M and embryo transfer, as well as BRCA screening and other treatment related costs including, RRSO, RRM and breast and ovarian cancer treatment costs were received from the Israeli ministry of health 2020 pricing list according to specified codes with conservative assumptions of health resources utilities. Elaborate costs used in the model, from payer perspective, are shown in supplementary Table 1, 2, 3, 4 and 5 for, ovarian cancer treatment, breast cancer treatment, IVF/PGT-M, BRCA positive patients follow-up and total costs, respectively. All costs were discounted at 3%.

### Probabilities

Model probabilities are presented in Table 1. The probability of being at the end of each arm of the Markovian model was calculated by multiplying the probabilities of events along the arms’ path. Stage distribution and Kaplan Meir survival curves for each stage were used to calculate mortality rates of subjects who developed breast cancer or ovarian cancer, based on the Surveillance, Epidemiology and End Results program data base (SEER) [13, 14]. Survival curves were extrapolated by fitting Weibull distribution using the Nelder-Mead Algorithm.

**Table 1:** Probabilities used in model

Probability	Description	Probability	Range assumed
P1	Probability of male newborn	0.5	
P2	Probability newborn is BRCA carrier	0.5	
P3	Probability that a non-carrier will experience ovarian cancer ^13^	0.0128	0.0005-0.0989
P4	Probability that a non- carrier will experience breast cancer^14^	0.13	0.11-0.14
P5	Probability that carrier will undergo RRSO ^20,21^	0.65	0.3-0.75
P6	Probability that BRCA carrier will undergo RRM ^25^	0.16	0.13-0.3
P7	Reduction in risk of ovarian cancer from RRSO ^26,27^	0.8	0.8-0.96
P8	Probability that BRCA carrier without RRSO will get OC ^28^	0.2987	0.24-0.35
P9	Reduction in risk of breast cancer from RRM and RRSO^27^	0.91	0.78-0.99
P10	Probability that a BRCA carrier without RRM will experience breast cancer ^28^	0.53	0.44-0.62
P11	Reduction in risk of breast cancer from RRSO ^22,24,29^	0.0	0.37-0.65
P12	Reduction in breast cancer risk from RRM without RRSO ^30^	0.91	0.62-0.98
P13	Probability of live newborn with IVF/PGD (1 cycles of fresh ET and 2 cycles of thawed ET)	0.84	0.7-0.9

*RRSO* Risk reduction salpingo-oophorectomy, *RRM* Risk reduction mastectomy, *IVF* In-vitro fertilization, *PGD* Pre-gestational diagnosis, *ET* Eembryo transfer

### Quality adjusted life years and incremental cost effectiveness ratio

Value of health benefits for each strategy (IVF/ PGT-M versus natural conception) were calculated using quality adjusted life years (QALY’s). QALY’s are calculated by multiplying the utility value associated with a given state of health by the number of years lived in that state, where QALY of one reflects 1 year lived in perfect health and QALY of zero represents death state. The incremental cost effectiveness ratio was then calculated by using the formula: (average cost IVF/ PGT-M – average cost natural conception) / (average QALY IVF/ PGT-M – average QALY natural conception). The incremental cost effectiveness ration (ICER) calculated, enabled to determine whether offering IVF/ PGT-M to BRCA positive families is cost effective or not, compared to willingness to pay threshold. An intervention was defined as cost effective if the ICER per QALY is between 1 and 3 times per capita gross national product (GNP). GNP in Israel is estimated at 42,160 USD, equivalent to 142,500 new Israeli Shekels (NIS) [15]. Interventions below 1 GNP per capita are considered very cost effective [16].

### Sensitivity analysis

One-way sensitivity analysis was conducted with all variables to evaluate model uncertainties. In addition, a probabilistic sensitivity analysis (Monte Carlo simulation) was conducted with all variables, using 100 trials, each included 10,000 couples.

## Results

Delivering a BRCA negative newborn after IVF/ PGT-M, compared to natural conception, is cost effective according to our model, with an ICER of 150,219 NIS per QALY gained, at a 3% discount rate compared to a willingness to pay threshold of 1–3 times Israeli GNP per capita, equivalent to 44,480 USD (Table 2).

**Table 2:** Cost effectiveness analysis of IVF/PGD versus natural conception for BRCA negative embryo selection

Strategy	Cost (NIS)	Incremental cost	QALY	Incremental QALY	ICER (NIS)
3% discount rate					
IVF/PGD	12,057		30.1359		
Natural conception	30,868	18,811	30.2611	0.1252	150,219
Not discounted (0% discount rate)					
IVF/PGD	49,767	0	77.7	0	
Natural selection	66,809	17,041	76.8	-0.866	-19,658

*IVF* In vitro-fertilization, *PGD* Preimplantation genetic diagnosis, *QALY* Quality adjusted life years, *ICER* Incremental cost effectiveness ration, *NIS* New Israeli Shekels

### Sensitivity analysis

**Fig. 2:**
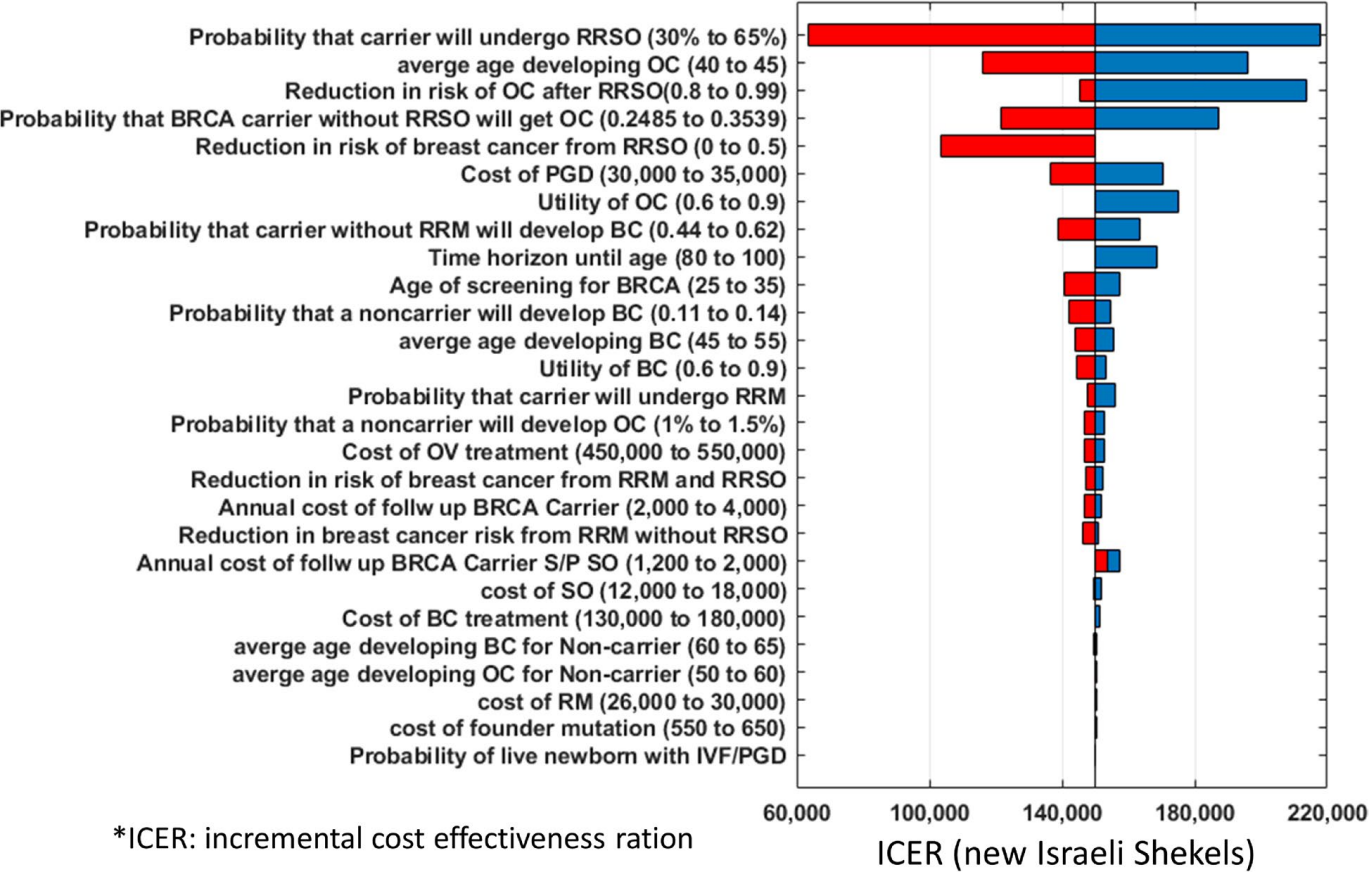
Tornado diagram, sensitivity analysis of influence of model parameters on incremental cost effectiveness ratio

In order to assess the effect of each parameter on the ICERS, a sensitivity analysis was conducted. Results are shown on a tornado diagram (Fig. 2). The most influential parameter that effects the ICER was found to be the discount rate, set at 3% in our model. As the money spent on IFV/ PGT-M is used at present, while screening strategies for BRCA carriers, risk reduction surgeries as well as breast and ovarian cancer treatments start many years later (beginning at age 30 years), discounting makes costs of future spending much lower than current values. Reducing the discount rate to zero resulted in a negative ICER of − 19,658 NIS, meaning that IVF/ PGT-M would be cost saving and not just cost effective.

Probabilistic sensitivity analysis (Monte Carlo simulation) was conducted with all variables, using 100 trials, each included 10,000 couples. At a willingness to pay threshold of 340,000 NIS (which are equivalent to 100,000 USD), in over 85% of simulations iterations IVF/ PGT-M compared to natural conception is cost effective (Acceptability curve, which shows the results of probabilistic sensitivity analysis and presents the relative cost-effectiveness as a function of the ICER, willingness to pay threshold, is shown in Supplementary Fig. 1).

## Discussion

In this cost effectiveness analysis, we found that at current pricing, with a 3% discount rate, IVF/ PGT-M for BRCA negative embryo selection is cost effective compared to natural conception, with an ICER of 150,219 NIS per QALY gained. This ICER is just short of being very cost effective, compared to a WTP threshold of one GNP per capita in Israel.

Advances in PGT-M can prevent passing of single gene disorders such as BRCA mutations to future generations by selecting a BRCA negative embryo. The prevalence of BRCA gene mutations reaches as high as 2.5% among Jews of Ashkenazi ancestry [17] and although offering IVF/ PGT-M on a large population scale may not be feasible, this technology can be an extremely useful solution for families with very high BRCA related morbidity or mortality. These families carry a heavy medical as well as psychological burden with a life-long fear of cancer as well as fear of passing this cancer potential to their descendants. Our model shows that IVF/PGT-M is a cost effective option that may be offered to selected patients. This option should be kept in mind of gynecologists, gynecologic oncologists, oncologist and any other physician involved with medical care of BRCA positive patients.

The sensitivity analysis showed that the most important factor impacting the ICER in our model is the discount rate. This is caused because the costs involved in IVF/ PGT-M are assumed to be spent at present while the money saved by preventing the birth of a BRCA positive offspring will only be seen within 30–40 years when screening and risk reduction surgeries will be implemented. Thus, the long projection period makes the yearly discount a very influential factor in the model. Reducing the discount rate to zero resulted in a negative ICER (− 19,658 NIS), making IVF/PGT-M cost saving. In a similar work, only recently published by Lipton et-al, a more conservative discount rate of 1.5% was assumed [18]. In this work, that was done on Canadian data, IVF/PGT-M was found to be cost effective for both BRCA1 and BRCA2 mutation carriers with an ICER of 14,242 USD and 12,893 USD, respectively. In our model the base line discount rate assumed was 3%, one that is used more frequently in cost effectiveness analysis in the United States and in most European countries [19].

The probability that a BRCA carrier would undergo RRSO also had a significant influence on the ICER calculated (tornado diagram, Fig. 2). The probability estimated in our model was 65%, based on previous data [20, 21], raising this probability would make the ICER significantly more cost effective. Presuming that IFV/PGT-M would be offered to families with high disease burden, we believe that the uptake of both RRSO and RM would be much higher among these families, making IFV/PGT-M even more cost effective. The least influential factor that effects the ICER was found to be the probability of a live, BRCA negative baby after IVF/PGT-m (tornado diagram, Fig. 2). In our model this was calculated to be 84% after one round of fresh embryo transfer and two more rounds of thawed embryo transfer for women under age 35. IVF/PGT-m success rates may undoubtedly differ between clinics, techniques, protocols, experience and expertise and these differences are reflected in different reports regarding IVF success rates. Yet our model shows that even a large overestimation or underestimation of IVF/PGT-m success rates has little effect on the ICER. Even doubling the percent of couples that would fail to deliver a BRCA negative baby through IVF/PGT-m and that would need to go back and conceive naturally after unsuccessful IVF/PGT-m from 16 to 30% was found to hardly effect the ICER calculated (Fig. 2).

Our work has several limitations. As corralation between BRCA gene mutation and other malignancies, such as prostate, gastrointestinal and pancreatic cancer is much weaker we decided not to include these in our model. The number of uncertanties that would arise in a model that would include many more malignancies would be, in our opinion, too large, making our theoretical model much weaker. Another major limitation that would need to be addressed as IVF and PGT-M technologies improve, making it more readily available for widespread use is the ethical justification of putting couples through a potentially dangerous medical procedure in order to avoid potential disease that would develop many years later such as ovarian cancer that is highly preventable with RRSO and breast cancer that is both preventable and highly curable for patients under tight follow-up. From a practical standpoint, the prevalence of BRCA in Israel, particularly among Jews of Askenazi origin, is as high as 2.5%, and offering IVF/ PGT-M on a large population scale might not be a feasible option. Saving this procedure to selected families with high disease burden or extreme cancer anxiety would be a more practical approach.

Our work has several advantages. In our model, IVF/ PGT-M strategy was found to be cost effective compared to natural conception. When calculating the costs of breast and ovarian cancer treatment, we assumed a very conservative use of health resource utilities. In addition, costs involved with male BRCA related malignancies were not included in our calculations, (mainly because the probabilities of these malignancies are much smaller). Had we taken these risks into consideration in our model, cost-effectiveness would likely to increase considerably. Another important point that underestimates the advantage of having IVF/ PGT-M, and would make this strategy more cost effective, is the elimination of the 50% chance of passing on the BRCA gene to second generations in the IVF/ PGT-M arm. In the natural conception arm, BRCA positive newborns would themselves have a 50% chance of passing the BRCA mutated gene to their offspring with the costs involved. Our model included the most up-to-date recommendations for breast and ovarian cancer treatments including the use of poly ADP ribose polymerase (PARP) inhibitors for the first line treatment of ovarian cancer, that was only recently approved for use in Israel, in BRCA positive patients. These are highly costly drugs that add considerably to cancer treatment costs. Most up-to-date data regarding the effect of RRSO on breast cancer risk was also included in our model. Previous data estimated a 50% reduction in risk of breast cancer as a result of RRSO. Newer data that used RRSO as a time dependent covariate did not find such an effect and in fact found no influence of RRSO on breast cancer risk [22–24]. A possible strategy that would further increase success rates of IVF/PGT-M, for couples who failed to produce a BRCA negative embryo would be to transfer a male embryo, regardless of its’ BRCA status. Using a male BRCA positive embryo for transfer would not prevent passing on the BRCA gene to the next generation, but would eliminate the risk of ovarian cancer, keep the chance of breast cancer (male breast cancer) very low while increasing the number of embryos available for transfer.

## Conclusion

IVF/PGT-M for BRCA positive parents versus natural conception at current pricing, with a 3% discount rate, is cost effective and should be offered to selected couples. Discount rate is the most influential parameter that effects the ICER.

## Supplementary Information

**Additional file 1: Supplementary Table 1**: Ovarian cancer diagnosis and treatment costs.

**Additional file 2: Supplementary Table 2**: Breast cancer diagnosis and treatment costs (All costs received from IMH* pricing list (1).

**Additional file 3: Supplementary Table 3**: Yearly BRCA carrier follow up costs (All costs received from IMH* pricing list [1] according to NCCN surveillance guidelines (2).

**Additional file 4: Supplementary Table 4**: IVF PGD costs used in model, according to Israeli ministry of health (IMH) pricing list^1^ (1 fresh round+ 2 thawed rounds)*.

**Additional file 5: Supplementary Table 5**: Total costs used in model.

**Additional file 6.** Acceptability curve.

## Data Availability

Not applicable (theoretical mathematical model).

## Abbreviations

IVF: In-vitro fertilization
PGT-M: Preimplantation genetic testing
ICSI: Intracytoplasmic sperm injection
RRSO: Risk reduction salpingo-oophorectomy
RRM: Risk reduction mastectomy
QALYs: Quality-adjusted life years
ICER: Incremental cost effectiveness ratio
GNP: Gross national product
NIS: New Israeli Shekels
