# Supplementary material for: Preimplantation genetic testing for BRCA gene mutation carriers: a cost effectiveness analysis
Source: Reprod Biol Endocrinol. 2021 Oct 8;19:153. doi: 10.1186/s12958-021-00827-9 (PMC8499576; doi:10.1186/s12958-021-00827-9)
Supplement: Supplementary file 1 — Additional file 1: Supplementary Table 1: Ovarian cancer diagnosis and treatment costs. [file 12958_2021_827_MOESM1_ESM.docx]

Supplementary Table 1: ovarian cancer diagnosis and treatment costs

(All costs received from IMH* pricing list (1)

|  | IMH code | Cost per service (NIS) | Number of services assumed in the model | Total cost per patient | remarks |
| --- | --- | --- | --- | --- | --- |
| BRCA founder mutation testing | J1311 | 632 | 1 | 632 | IMH pricing list |
| CT scan | L1039 | 711 | 5 | 3,555 | IMH pricing list |
| Pathology examination | 88305 | 217 | 1 | 217 | IMH pricing list |
| Immunohystochemistry staining | L0564 | 2,070 | 1 | 2,070 | IMH pricing list |
| CT guided biopsy | 77012 | 5,699 | For 70% of patients | 3,989 | IMH pricing list |
| Oncology clinic check up | L9244 | 153 | 10 | 1530 | IMH pricing list |
| Gynecology clinic check up | L9255 | 153 | 10 | 1530 | IMH pricing list |
| Serum CA-125 levels | 86304 | 48 | 10 | 480 | IMH pricing list |
| Preoperative checkup | 99223 | 987 | 1 | 987 | IMH pricing list |
| Hospital admission per day: 1^st^ 3 days | G00H2 | 3,271 | 15 | 49,065 | IMH pricing list |
| Hospital admission per day 4^th^ day on | G00H4 | 2,844 | 20 | 56,880 | IMH pricing list |
| Debulking surgery (coded as Radical Hysterectomy) | G00T9 | 28,500 | 1 | 28,500 | IMH pricing list |
| Pelvic US | L6856 | 398 | 5 | 1,990 | IMH pricing list |
| Oncology day care treatment | L6550 | 1,618 | 20 | 32,360 | IMH pricing list |
| Neulastim (6 mg) | 4971 | 3,469/6mg  Need 6 mg | 2 | 6,938 | IMH pricing list |
| Akynzeo (1 dose) | 7798 | 328/1 pack | 6 | 1,968 | IMH pricing list |
| 1^st^ line Carboplatin 450 mg | 7445 | 407(450 mg)  Need 700 mg/cycle | 6  633 NIS /cycle *6 cycles | 3,798 | IMH pricing list |
| 1^st^ line Paclitaxel 30mg | 8349 | 1473/300 mg  Need 300mg | 6  1473 NIS /cycle*6 cycles | 8,838 | IMH pricing list |
| 2^nd^ line chemotherapy +supportive care (70% of patients) |  |  | (3,798+8,838+1,968+,3798)*0.7 | 12,881 | Assuming 70% of patients relaps(2–4) |
| Bevacizumab | 4933 | 4,965/400 mg  Need 525 mg/cycle | 17  525mg =6,516  (6,516*17 cycles)*40% of patients | 44,312 | 20% stage 4 + 20% suboptimal debulking(5,6) |
| BRCA somatic mutation testing | J1312 | 4,093 | 1 | 4,093 |  |
| Lynparza as maintenance therapy for first line BRCA positive patients | 7618 | 600mg/day for 24 months | 25,587/month*24 months for 30% BRCA positive patients, 28% dose reduction | 184,230 | Average 24 months treatment for 30% BRCA positive (7) |
| Average terminal care for cancer patients (last 6 months) |  | 63,586 | 1 (for 70% of patients) | 44,510 | Assuming all relapsed patients succumb to their disease (8) |
| Total cost |  |  |  | 495,353 |  |

IMH: Israeli ministry of health, NIS: New Israeli shekels

1. Ministry of Health pricing list, 2020 [Internet]. 2020 [cited 2020 Oct 15]. Available from: https://www.health.gov.il/Subjects/Finance/Taarifon/Pages/PriceList.aspx

2. Bruchim I, Jarchowsky-Dolberg O, Fishman A. Advanced (>second) line chemotherapy in the treatment of patients with recurrent epithelial ovarian cancer. Eur J Obstet Gynecol Reprod Biol [Internet]. Elsevier Ireland Ltd; 2013;166:94–8. Available from: http://dx.doi.org/10.1016/j.ejogrb.2012.10.003

3. Kessous R, Laskov I, Abitbol J, Bitharas J, Yasmeen A, Salvador S, et al. Clinical outcome of neoadjuvant chemotherapy for advanced ovarian cancer. Gynecol Oncol [Internet]. Elsevier Inc.; 2017;144:474–9. Available from: http://dx.doi.org/10.1016/j.ygyno.2016.12.017

4. Houben E, van Haalen HGM, Sparreboom W, Overbeek JA, Ezendam NPM, Pijnenborg JMA, et al. Chemotherapy for ovarian cancer in the Netherlands: a population-based study on treatment patterns and outcomes. Med Oncol [Internet]. Springer US; 2017;34:50. Available from: http://link.springer.com/10.1007/s12032-017-0901-x

5. Oza AM, Cook AD, Pfisterer J, Embleton A, Ledermann JA, Pujade-Lauraine E, et al. Standard chemotherapy with or without bevacizumab for women with newly diagnosed ovarian cancer (ICON7): Overall survival results of a phase 3 randomised trial. Lancet Oncol. 2015;16:928–36.

6. Israeli Ministry of Health, recommendations for Bevacizumab use [Internet]. 2017. Available from: https://www.health.gov.il/hozer/mk01_2017.pdf

7. Ray-Coquard I, Pautier P, Pignata S, Pérol D, González-Martín A, Berger R, et al. Olaparib plus Bevacizumab as First-Line Maintenance in Ovarian Cancer. N Engl J Med [Internet]. Massachussetts Medical Society; 2019 [cited 2020 Sep 20];381:2416–28. Available from: http://www.nejm.org/doi/10.1056/NEJMoa1911361

8. Bentor N RSTTBR. Use of end of life care, quality and costs of patients that have died from metastatic cancer [Internet]. Myers-JDC-Brookdale Inst. 2014 [cited 2020 Sep 26]. Available from: https://brookdale.jdc.org.il/wp-content/uploads/2018/01/Quality_and_cost_Report_HEB.pdf
