# Supplementary material for: Preimplantation genetic testing for BRCA gene mutation carriers: a cost effectiveness analysis
Source: Reprod Biol Endocrinol. 2021 Oct 8;19:153. doi: 10.1186/s12958-021-00827-9 (PMC8499576; doi:10.1186/s12958-021-00827-9)
Supplement: Supplementary file 2 — Additional file 2: Supplementary Table 2: Breast cancer diagnosis and treatment costs (All costs received from IMH* pricing list (1). [file 12958_2021_827_MOESM2_ESM.docx]

Supplementary Table 2: Breast cancer diagnosis and treatment costs (All costs received from IMH* pricing list (1)

|  | IMH code | Cost per service (NIS) | Number of services assumed in the model | Total cost per patient | Remarks |
| --- | --- | --- | --- | --- | --- |
| Mammography | 77056 | 279 | 1 | 279 | IMH pricing list |
| Breast US | 76645 | 277 | 1 | 277 | IMH pricing list |
| Imaging guided Breast biopsy | 19102 | 2,504 | 1 | 2,504 | IMH pricing list |
| Pathology examination | 88305 | 217 | 2 | 434 | IMH pricing list |
| Immunohystochemistry staining | L0564 | 2,070 | 2 | 4,140 | IMH pricing list |
| Breast cancer hormonal receptor testing | 84233 | 741 | 2 | 1,482 | IMH pricing list |
| Molecular profiling (for ER +) |  | 12,000 | 1 | 12,000 | Oncotype Dx® |
| Breast surgeon follow up | L9266 | 153 | 5 | 765 | IMH pricing list |
| Preop checkup | 99223 | 987 | 1 | 987 | IMH pricing list |
| Lumpectomy/mastectomy | G0051 | 19,671 | 1 | 19,671 | IMH pricing list |
| Hospital admission 1^st^ 3 days | G00H2 | 3,271 | 6 | 19,626 | IMH pricing list |
| Hospital admission 4^th^ day onwards | G00H4 | 2,844 | 4 | 11,376 | IMH pricing list |
| Imaging PET-FDG | 78813 | 5,017 | 1  For 35% of locally advanced staging | 1,755 | IMH pricing list |
| Oncology clinic check up | L9244 | 153 | 10 | 1,530 | IMH pricing list |
| Radiotherapy simulation | 77280 | 2,593 | 1  75% of patients | 1,944 | IMH pricing list |
| Radiotherapy single fraction | 77401 | 512/fraction (need 20 frc) | 20  75% of patients  (20*502)*0.75 | 7,680 | For 75% of patients (2–4) |
| Paclitaxel 80mg/m2 (45%) | 6130 | 258/30 mg need 140 mg | 12  (1,204/cycle*12 cycles)*0.45 | 6,501 | For 45% of patients (4,5) |
| Cyclophosphamide (endoxan) 600 mg/m2 (45%) | 4081 | 40/500 mg (need 1050 mg) | 4  (84/cycle*4cycles)*0.45 | 151 | For 45% of patients (4,5) |
| Adriamycin 60 mg/m2 (45%) | 7926 | 482/200 mg  (need 105 mg) | 4  (253/cycle*4cycles)*0.45 | 455 | For 45% of patients (4,5) |
| Herceptin trastuzumab (15% of patients) | 4915 | 5,568/440mg  (need 6mg/kg) | 17  (17 cycles*5,568)*0.15 | 14,198 | For 15% of patients (4,5) |
| Pertuzumab (Perjeta) (15%) | 7116 | 12,460/420mg  need 420mg | 6  (6 cycles*14,192)*0.15 | 11,214 | For 15% of patients (4,5) |
| neulastim | 4971 | 3,469/6mg  Need 6 mg | 4 | 13,876 | IMH pricing list |
| Akynzeo | 7798 | 328/1 pack | 4 | 1,312 | IMH pricing list |
| Olaparib | 8454 | 26,035/months  600mg/day | 26,035  For 7 months  For 5% BRCA positive  For 36% metastatic disease | 3,280 | For 5% of BRCA positive patients with 36% metastatic disease (6,7) |
| Tamoxifen | 6527 | 35/month | For 5 years  75% of patients | 1,575 |  |
| Oncology day care treatment | L6550 | 1,618 | 16 | 25,888 | IMH pricing list^1^ |
| Average terminal care for cancer patients (last 6 months) |  | 63,586 | 15% of patients | 9,538 | Assumed for 15% of patients with advanced disease(8) |
| Total cost |  |  |  | 174,438 |  |

*IMH: Israeli ministry of health, NIS: new Israeli Shekels

1. Ministry of Health pricing list, 2020 [Internet]. 2020 [cited 2020 Oct 15]. Available from: https://www.health.gov.il/Subjects/Finance/Taarifon/Pages/PriceList.aspx

2. Metzger-Filho O, Sun Z, Viale G, Price KN, Crivellari D, Snyder RD, et al. Patterns of recurrence and outcome according to breast cancer subtypes in lymph node-negative disease: Results from international breast cancer study group trials VIII and IX. J Clin Oncol. 2013;

3. Zeidan YH, Habib JG, Ameye L, Paesmans M, de Azambuja E, Gelber RD, et al. Postmastectomy Radiation Therapy in Women with T1-T2 Tumors and 1 to 3 Positive Lymph Nodes: Analysis of the Breast International Group 02-98 Trial. Int J Radiat Oncol Biol Phys. Elsevier Inc.; 2018;101:316–24.

4. Senkus E, Kyriakides S, Ohno S, Penault-Llorca F, Poortmans P, Rutgers E. Primary breast cancer: ESMO Clinical Practice clinical practice guidelines. Ann Oncol. 2015;26.

5. Israeli cancer registry. Breast cancer in Israel, 2018 [Internet]. [cited 2019 Sep 20]. Available from: https://www.health.gov.il/PublicationsFiles/breast_cancer_SEPT2018.pdf

6. Ministry of health publication. Health services basket, 2020 [Internet]. 2020 [cited 2020 Sep 25]. Available from: https://www.health.gov.il/hozer/mk03_2020.pdf

7. Robson M, Im SA, Senkus E, Xu B, Domchek SM, Masuda N, et al. Olaparib for metastatic breast cancer in patients with a germline BRCA mutation. N Engl J Med [Internet]. Massachussetts Medical Society; 2017 [cited 2020 Sep 25];377:523–33. Available from: https://pubmed.ncbi.nlm.nih.gov/28578601/

8. Bentor N RSTTBR. Use of end of life care, quality and costs of patients that have died from metastatic cancer [Internet]. Myers-JDC-Brookdale Inst. 2014 [cited 2020 Sep 26]. Available from: https://brookdale.jdc.org.il/wp-content/uploads/2018/01/Quality_and_cost_Report_HEB.pdf
