# Supplementary material for: Preimplantation genetic testing for BRCA gene mutation carriers: a cost effectiveness analysis
Source: Reprod Biol Endocrinol. 2021 Oct 8;19:153. doi: 10.1186/s12958-021-00827-9 (PMC8499576; doi:10.1186/s12958-021-00827-9)
Supplement: Supplementary file 3 — Additional file 3: Supplementary Table 3: Yearly BRCA carrier follow up costs (All costs received from IMH* pricing list [1] according to NCCN surveillance guidelines (2). [file 12958_2021_827_MOESM3_ESM.docx]

Supplementary Table 3: Yearly BRCA carrier follow up costs (All costs received from IMH* pricing list (1) according to NCCN surveillance guidelines(2)

|  | IMH code | Cost per service (NIS) | Number of services assumed in the model (per year) | Total cost per patient |
| --- | --- | --- | --- | --- |
| Breast US | 76645 | 277 | 1 | 277 |
| Breast MRI | 77059 | 2,068 | 1 | 2,068 |
| Breast surgeon follow up | L9266 | 153 | 2 | 306 |
| Hormone replacement therapy after RRSO | 4568 | 60/month | 12 for 50% of patients(3)  For 10 years | 360 |
| Total cost before RRSO |  |  |  | 2,651 |
| After RRSO |  |  |  | 2,753 |

IMH: Israeli ministry of health, NIS: New Israeli shekels

1. Ministry of Health pricing list, 2020 [Internet]. 2020 [cited 2020 Oct 15]. Available from: https://www.health.gov.il/Subjects/Finance/Taarifon/Pages/PriceList.aspx

2. NCCN Clinical Practice Guidelines in Oncology [Internet]. [cited 2019 Dec 14]. Available from: https://www.nccn.org/professionals/physician_gls/default.aspx#detection

3. Johansen N, Liavaag AH, Iversen OE, Dørum A, Braaten T, Michelsen TM. Use of hormone replacement therapy after risk-reducing salpingo-oophorectomy. Acta Obstet Gynecol Scand [Internet]. Wiley-Blackwell; 2017 [cited 2020 Sep 20];96:547–55. Available from: https://pubmed.ncbi.nlm.nih.gov/28236297/
