# Supplementary material for: Preimplantation genetic testing for BRCA gene mutation carriers: a cost effectiveness analysis
Source: Reprod Biol Endocrinol. 2021 Oct 8;19:153. doi: 10.1186/s12958-021-00827-9 (PMC8499576; doi:10.1186/s12958-021-00827-9)
Supplement: Supplementary file 4 — Additional file 4: Supplementary Table 4: IVF PGD costs used in model, according to Israeli ministry of health (IMH) pricing list1 (1 fresh round+ 2 thawed rounds)*. [file 12958_2021_827_MOESM4_ESM.docx]

Supplementary Table 4: IVF PGD costs used in model, according to Israeli ministry of health (IMH) pricing list^1^ (1 fresh round+2 thawed rounds)*

|  | IMH code | Cost (Israeli shekels) | Remark | Total costs |
| --- | --- | --- | --- | --- |
| IVF first stage | L8970 | 4,509 | 100% | 4,433 |
| IVF second stage | L8972 | 5,434 | 100% | 5,343 |
| Fertility medication for ovum pick-up (1 cycle):  Gonal F (900IU)  Cetrotide (0.25mg)  Ovitrelle (250mcg)  Estrofem (2mg)  Utrogestan (200mg) | 6544  4054  4917  2694  4728 | 1,583*3 pens  1,398*2 packs  153  21*3 packs  35*14 packs | 225 IU/day | 8251 |
| ICSI | 89280 | 3,952 | 82% | 3,240 |
| Preimplantation diagnosis | 89290 | 11,404 | 49% | 5,588 |
| Embryo vitrification | 89258 | 798 | 100% | 798 |
| Thawed embryo transfer (first round) | 58974 | 4,688 | 54.2% | 2,540 |
| Fertility medication thawed embryo transfer  Estrofem (2mg)  Utrogestan (200mg) | 2694  4728 | 21*3 packs  35*14 packs | 54.2% | 300 |
| Thawed embryo transfer (second round) | 58974 | 4,688 | 29% | 1,360 |
| Fertility medication thawed embryo transfer  Estrofem (2mg)  Utrogestan (200mg) | 2694  4728 | 21*3 packs  35*14 packs | 29% | 160 |
| Total IVF PGD costs |  |  |  | 32,013 |

1. Ministry of Health pricing list, 2019, [https://www.health.gov.il/Subjects/Finance/Taarifon/Pages/PriceList.aspx. Published 2019](https://www.health.gov.il/Subjects/Finance/Taarifon/Pages/PriceList.aspx.%20Published%202019). Accessed September, 2019.
