# Supplementary material for: Preimplantation genetic testing for BRCA gene mutation carriers: a cost effectiveness analysis
Source: Reprod Biol Endocrinol. 2021 Oct 8;19:153. doi: 10.1186/s12958-021-00827-9 (PMC8499576; doi:10.1186/s12958-021-00827-9)
Supplement: Supplementary file 5 — Additional file 5: Supplementary Table 5: Total costs used in model. [file 12958_2021_827_MOESM5_ESM.docx]

Supplementary Table 5: Total costs used in model

|  | IMH* code | Cost (Israeli shekels) | Remark |
| --- | --- | --- | --- |
| IFV PGD treatment costs (1 fresh and 2 thawed cycles) |  | 32,013 | Calculated (supplementary table 4) |
| Risk reduction salpingo oophorectomy | G0231 | 16,034 | Ministry of Health pricing list ^25^ |
| Risk reduction mastectomy | G0045 | 29,510 | Ministry of Health pricing list ^25^ |
| Ovarian cancer diagnosis and treatment |  | 495,353 | Calculated (supplementary table 1) |
| Breast cancer diagnosis and treatment |  | 174,438 | Calculated (supplementary table 2) |
| Total cost before RRSO |  | 2,651 | Calculated (supplementary table 3) |
| After RRSO |  | 2,753 | Calculated (supplementary table 3) |

*IMH: Israeli ministry of health, IVF: in-vitro fertilization, PGD: pre-gestational diagnosis, RRSO: risk reduction salpingo-oophorectomy
