# Supplementary figures and images for: Preimplantation genetic testing for BRCA gene mutation carriers: a cost effectiveness analysis
Source: Reprod Biol Endocrinol. 2021 Oct 8;19:153. doi: 10.1186/s12958-021-00827-9 (PMC8499576; doi:10.1186/s12958-021-00827-9)

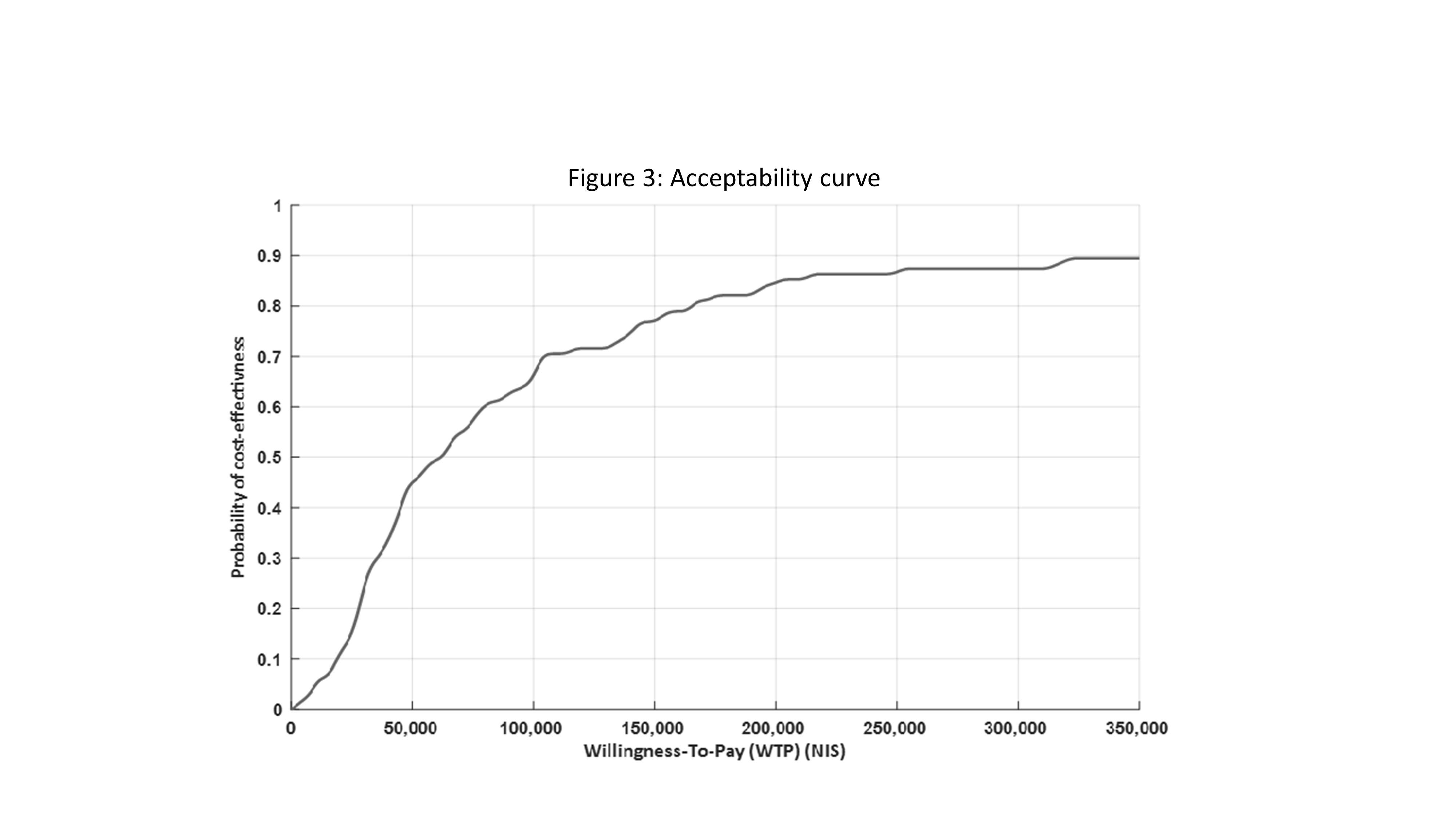

Supplement: Supplementary file 6 — Additional file 6. Acceptability curve. [file 12958_2021_827_MOESM6_ESM.tif]
